# Photolysis of Low-Brominated Diphenyl Ethers and Their Reactive Oxygen Species-Related Reaction Mechanisms in an Aqueous System

**DOI:** 10.1371/journal.pone.0135400

**Published:** 2015-08-14

**Authors:** Mei Wang, Huili Wang, Rongbo Zhang, Meiping Ma, Kun Mei, Fang Fang, Xuedong Wang

**Affiliations:** 1 Key Laboratory of Watershed Science and Health of Zhejiang Province, Wenzhou Medical University, Wenzhou 325035, China; 2 College of Life Sciences, Wenzhou Medical University, Wenzhou 325035, China; University of Illinois at Chicago, UNITED STATES

## Abstract

To date, no report was concerned with participation of reactive oxygen species in waters during photolysis of low-brominated diphenyl ethers (LBDEs). Herein, we found that electron spin resonance (ESR) signals rapidly increased with increasing irradiation time in the solution of LBDEs and 4-oxo-TMP solutions. But this phenomenon did not occur in the presence of NaN_3_ (^1^O_2_ quencher) demonstrating generation of ^1^O_2_ in process of LBDEs photolysis. The indirect photolytic contribution rate for BDE-47 and BDE-28 was 18.8% and 17.3% via ^1^O_2_, and 4.9% and 6.6% via ·OH, respectively. Both D_2_O and NaN_3_ experiments proved that the indirect photolysis of LBDEs was primarily attributable to ^1^O_2_. The bimolecular reaction rate constants of ^1^O_2_ with BDE-47 and BDE-28 were 3.12 and 3.64 × 10^6^ M^-1^ s^-1^, respectively. The rate constants for BDE-47 and BDE-28 (9.01 and 17.52 × 10^−3^ min^-1^), added to isopropyl alcohol, were very close to those (9.65 and 18.42 × 10^−3^ min^-1^) in water, proving the less indirect photolytic contribution of ·OH in water. This is the first comprehensive investigation examining the indirect photolysis of LBDEs in aqueous solution.

## Introduction

Polybrominated diphenyl ethers (PBDEs), used as an additive in brominated flame retardants, have been widely used in textile, electronic, food and home furnishing products [1–3]. There are 209 kinds of congeners, which are divided into low-brominated diphenyl ethers (LBDEs, containing one to six bromine atoms) and high-brominated diphenyl ethers (HBDEs, containing seven to ten bromine atoms). HBDEs can be transformed into LBDEs through various environmental reactions, especially by photolytic processes, leading to LBDEs being the most frequently detected congeners in real-world environmental matrices [4,5]. Among LBDEs, the predominant congeners are BDE-153, BDE-47 and BDE-28, which account for approximately 70–80% of total PBDEs in general people and the exposure of infants via breast feeding in Beijing [6]. In recent years, LBDE exposure in human breast milk, animal tissues and bloods have been reported in many studies [6,7]. Schröter-Kermani et al. [8] reported that the concentration of BDE-47 in milk samples from the USA had a median concentration of 18 μg kg^-1^ lipid. Concentrations of tetra- to hexa-brominated congeners in freshwater fish fillets in the USA ranged from <5 to 47,900 μg kg^-1^ (lipid basis) [9]. LBDEs are more toxic and bioaccumulative than HBDEs [10,11]. Chronic exposure of LBDEs to organisms can lead to liver tumors, developmental neurotoxicity and thyroid dysfunctions [12]. LBDEs depress thyroid development as well as the long-term learning and memory capabilities in adult rats [13,14].

Although LBDEs can undergo anaerobic and aerobic microbial degradation [15,16], the microbial degradation pathway is slow and inefficient. In contrast, photolysis by sunlight is generally considered the major pathway for LBDEs degradation in the environment [17], especially direct photolysis resulting from absorbance of the solar spectrum in the λ>290 nm range [11]. The current interpretation of these photolytic processes is exclusively based on the direct excitation of PBDEs to form singlet or triplet excited states that undergo C-Br cleavage of PBDEs (Eqs 1 and 2, where “*” refers to the excited state, and superscripts “1” and “3” refer to singlet and triplet states, respectively) [18,19].

PBDE+hv→P1BDE*→P3BDE* (1)

P1BDE*(P3BDE*)→debromination (2)

Fang et al. (2008) investigated the photolysis of five individual LBDEs (BDE-28, -47, -99, -100, and -153) in hexane and confirmed consecutive reductive debromination as the main degradation mechanism. Kuivikko et al. [17] predicted the photolytic half-lives in the mixing layer of the Baltic Sea and Atlantic Ocean using the photolytic quantum yields of BDE-47 and -99 in isooctane. In general, previous studies of LBDE photolysis were mostly performed in organic solvents or water-organic solvents [11,20–22], little data are available for natural waters because of the high hydrophobicity of LBDEs. However, the solvent has a strong effect on LBDE photolysis preventing direct extrapolation of results to aqueous environments. Eriksson et al. [11] made a tentative determination of photolytic kinetics of LBDEs in aqueous solutions containing 1% ethanol, but they inferred that the results were poor and that“due to its extremely low water solubility, photolytic experiments of LBDEs in the aqueous phase are practically impossible.” Suh et al. [18] confirmed that radical yields for PBDEs dissolved in tetrahydrofuran, dimethylformamide and toluene were about 9-, 4-, and 7-fold higher, respectively, than radical yields from the irradiated solvent alone. However, indirect photo-transformation of LBDEs with reactive oxygen species (ROS) remains uncertain in the aqueous phase. In organic solvents, reactive species such as ·OH and ^1^O_2_ will be more likely scavenged by the organic solvent that is more abundant and reactive than the target pollutant, and thus it may underestimate the power of ROS to degrade PBDEs in organic solvents. As a result, the indirect phototransformation of LBDEs with ROS requires rigorous investigation in aqueous environments in order to elucidate their real-world environmental behavior.

The main objective of this study was to elucidate the photolytic kinetics of LBDEs (BDE-47 and BDE-28) at concentrations representative of those observed in natural waters (ng L^-1^) and to investigate the related ROS-participating mechanisms. To achieve these objectives, the direct photolytic rate constant and quantum yield were measured for each single LBDE congener, and the indirect photolytic contribution due to *OH and ^1^O_2_ participation was appraised in natural waters. Additionally, an electron spin resonance (ESR) technique was employed to verify the participation of ROS. Furthermore, the interaction between photolysis of LBDEs and HBDEs was investigated to determine their optimal ratio for rapid photodegradation of LBDEs. To the best of our knowledge, this is the first comprehensive investigation examining the direct and indirect photolysis of LBDEs in aqueous solution. These results provide a theoretical foundation to explain mechanisms contributing to the high abundance of LBDEs, such as BDE-47 and BDE-28, in natural waters and for developing an optimal pathway for enhancing degradation of LBDEs in aquatic environments.

## Materials and Methods

### Reagents and chemicals

The following each single PBDE congener (at 50 μg mL^-1^ in isooctane) was purchased from Accustandard (New Haven, CT, USA): 2,4,4^/^-tribrominated diphenyl ether (BDE-28), 2,2^/^,4,4^/^-tetrabrominated diphenyl ether (BDE-47), 2,2^/^,4,4^/^,5-pentabrominated diphenyl ether (BDE-99), 2,2^/^,4,4^/^,5,5^/^-hexabrominated diphenyl ether (BDE-153), 2,2^/^,4,4^/^,5,6^/^- hexabrominated diphenyl ether (BDE-154), and 2,2^/^,3,4,4^/^,5,6-heptabrominated diphenyl ether (BDE-183). Sodium azide (NaN_3_, 99.5%), Rose Bengal (RB, 93%) and furfuryl alcohol (FFA, 98%) were purchased from Sigma-Aldrich. Pyridine, *p*-nitroanisole (PNA) and isopropyl alcohol (IPA) were obtained from Aladdin. 2,2,6,6-tetramethyl-4-piperidone (TEMP, 95%, Sigma Aldrich) and 5,5-dimethyl-1-pyrroline-N-oxide (DMPO, 97%, Sigma Aldrich) were stored at -20°C and used as the spin traps for ^1^O_2_ and ·OH/O_2_^·^, respectively. HPLC-grade acetonitrile and methanol were obtained from Merck Company (Darmstadt, Germany). All other chemical reagents were of analytical grade. Ultrapure water (18 MΩ) was obtained from a Millipore Milli-Q water system.

### Photolytic experiments

Photolytic experiments were performed in a photochemical reactor (model BL-GHX-V, Shanghai BiLon Corporation, Shanghai, China). Water-refrigerated 300 W mercury and xenon lamps equipped with 290 nm cutoff filters were employed to provide UV-Vis irradiation (λ>290 nm). The simulated sunlight and light intensity in the center of the reactive solutions within the photochemical reactor were 10.6 and 1.5 mW/cm^2^, respectively (S1 Fig).

To study the photolytic mechanisms of PBDE degradation in water, the stock solutions of LBDEs and HBDEs (20 mg L^-1^) were prepared in acetonitrile. A 35 mL volume with an initial concentration of 20 μg L^-1^ was prepared by diluting the stock solution with ultrapure water into a quartz tube. All photolytic experiments were performed in triplicate, and a corresponding dark control was prepared by wrapping the quartz tube with aluminum foil to protect from light. During the irradiation experiments, aliquots (5 mL) of the sample were transferred into a glass test tube at given time intervals for dispersive liquid-liquid microextraction (DLLME). The quantum yield, representing the fraction of molecules absorbing a photon that are transformed/degraded, was determined using the established *p*-nitroanisole (PNA, 60μM) with pyridine (6 mM) system. NaN_3_ (10 mM, quenchers of ^1^O_2_) and IPA (100 mM, quenchers of ·OH) were added to the photoreaction solutions to investigate indirect photolytic mechanisms [23].

### Dispersive liquid-liquid microextraction (DLLME) procedures

A 5.0 mL aliquot containing PBDEs was placed into a 15 mL screw cap glass test tube with conical bottom (S2 Fig). A mixture of 1.0 mL acetonitrile (dispersive solvent) and 22.0 μL 1,1,2,2-tetrachloroethane (extraction solvent) was injected rapidly into the sample with a 2 mL syringe. The other centrifugation and retrieving procedures were referred to methods reported by Liu et al. [24,25].

### Determination of reaction rates between ^1^O_2_ and LBDEs

To determine the ^1^O_2_ bimolecular reaction rate constants, 10 μM RB (a ^1^O_2_ sensitizer) was added to solutions containing 20 μg L^-1^ LBDEs (BDE-47 or BDE-28) and 200 μM FFA in ultrapure water under 300 W mercury lamp irradiation. The solutions were photolyzed in the solar simulator equipped with a 420 nm cutoff filter to limit the direct photolysis of LBDEs [23,26]. Aliquots (0.5 mL) of the solution were extracted at time intervals to detect the FFA concentration by HPLC as described below. The LBDE photolytic concentrations were analyzed by GC after they were extracted by liquid-liquid extraction using n-hexane as the extracting agent.

### Instrumental analyses

#### HPLC analysis

PNA and FFA were quantified using an Agilent HPLC-1260 system with a XDB-C_18_ column (5 μm, 150 mm × 4.6 mm) and UV/Vis detector. The optimized mobile phase for PNA was 50% acetonitrile-50% H_2_O, the flow rate was 0.8 mL min^-1^, and the detector wavelength was set at 300 nm. For FFA determination, the mobile phase was 20% acetonitrile-80% H_2_O with a flow rate of 0.8 mL min^-1^ and the detector wavelength was set at 218 nm.

#### Determination of PBDEs by GC

PBDE analysis was performed using an Agilent 7890 GC (Agilent Technologies, Wilmington, DE, USA) equipped with a micro-electron capture detector (μECD). The GC system was coupled to a HP-5 capillary column (30 m × 0.32 mm I.D., 0.25 μm film thickness, Agilent). Sample injections were performed in the splitless mode using an injection temperature of 290°C. The oven temperature was initially held at 110°C for 3 min, increased to 250°C for 5 min at 30°C min^-1^, and thereafter raised at 30°C min^-1^ to 300°C, which was held for 3 min. Nitrogen (purity 99.999%) was employed as the carrier gas at a constant flow rate of 1.5 mL min^-1^, and the split flow was set at 60 mL min^-1^.

#### Electron spin resonance (ESR) measurements

ESR signals for ^1^O_2_ and ·OH trapped by TEMP and DMPO were recorded on a Bruker A300 spectrometer (Bruker, Germany) equipped with a 150 W mercury lamp as the irradiation light source. The setting of the ESR spectrometer was as follows: microwave frequency, 9.85 kHz; microwave power, 20.20 mW; modulation frequency, 100 kHz; and center field, 3514.66 G.

#### UV-Vis absorption spectra

The absorption spectra of LBDEs in aqueous solution were acquired on a Shimadzu UV-2600 spectrophotometer (Shimadzu Co. Japan).

## Results

### DLLME-GC determination of LBDEs and HBDEs

The optimal DLLME conditions for determination of LBDEs were 1.0 mL acetonitrile as disperser solvent, 22.0 μL 1,1,2,2-tetrachloroethane as extraction solvent, 30 s hand-shaking at ambient temperature and without salt addition. Under these conditions, the relative fortified recoveries ranged from 87.0 to 107.6% for BDE-28, -47, -99, -153, -154, and -183. Enrichment factors reached up to 268–305 and linearity was observed in the range of 0.1–50 μg L^-1^. A repeatability study was carried out by extracting the spiked water samples at a concentration level of 20 μg L^-1^, and the relative standard deviations varied between 3.8 and 6.3% (n = 5). The limits of detection (LOD), based on a signal-to-noise ratio (S/N) of 3, were in the range of 8.1–28.2 ng L^-1^ (S1 Table). These results indicate that this DLLME method can be successfully applied to the determination of LBDEs and HBDEs in environmental water samples.

### Photolytic kinetics of LBDEs

The photolytic rate constants (*k*) increased with increasing substitution of bromine atoms on the benzene ring of PBDEs under 300 W xenon lamp irradiation (Table 1). The *k* values for hexabromodiphenyl ether (BDE-153 and BDE-154) were about 2.5-fold greater than that for tetrabromodiphenyl ether (BDE-47). The number of substituted bromine atoms in the benzene ring can affect the absorption wavelength of congeners, and the less bromine atoms congeners possess, the lower their ability to absorb radiation (BDE-153 or BDE-154, λ_max_ = 297; BDE-47, λ_max_ = 291) [11]. The different number of substituted bromine atoms lead to different molecular structures and physicochemical properties of congeners. The highest occupied molecular orbital energy (E_HOMO_) increases as the number of bromine atoms increases, which lead to an increase of the debromination rate (BDE-154, E_HOMO_ = -9.534 eV; BDE-153, E_HOMO_ = -9.400 eV; BDE-99, E_HOMO_ = -9.338 eV; BDE-47, E_HOMO_ = -9.329 eV) [27]. In the BDE-153 photolytic process, BDE-99 and BDE-47 were observed as photolytic products (S3 Fig). Similarly, BDE-47 was the main photolytic product of BDE-154, as previously reported [27].

**Table 1:** The estimated half-lives and rate constants for LBDEs under 300 W xenon lamp irradiation (λ> 290 nm) in water.

Parameters	BDE-154	BDE-153	BDE-99	BDE-47
Rate constants (*k*, *h* ^*-1*^)	12.8×10^−2^	11.0×10^−2^	7.80×10^−2^	4.63×10^−2^
Half-life (*t*_*1/2*_, h)	5.38	6.32	8.88	14.96
Coefficient of correlation (*R* ^*2*^)	0.9993	0.9996	0.9945	0.9962

**Fig 1:**
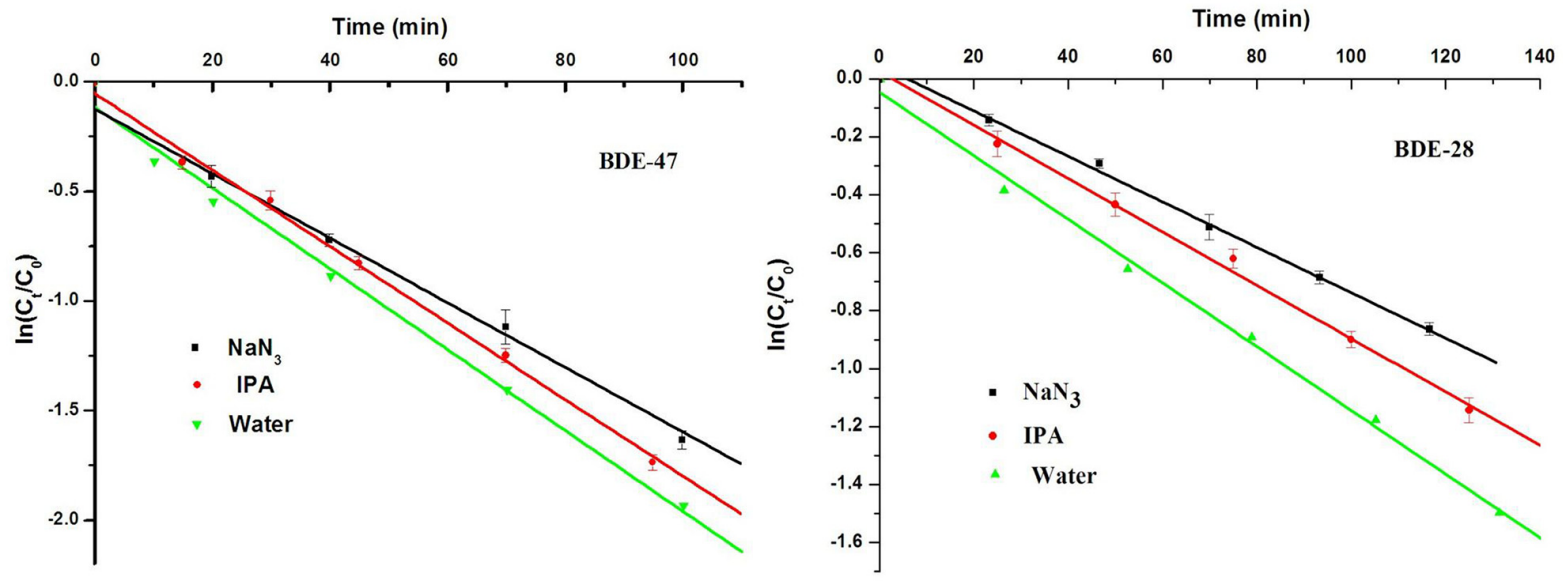
Effects of NaN_3_ (10 mM) and isopropyl alcohol (100 mM) photolytic kinetics of BDE-47 and BDE-28 in ultrapure water under 300 W mercury lamp irradiation (λ> 290 nm).

Similarly, the photolysis of BDE-47 and BDE-28 followed pseudo-first-order kinetics under under 300 W mercury lamp irradiation (λ>290 nm) with rate constants of 18.4 ×10^−3^ (R^2^ = 0.9909) and 9.65 ×10^−3^ min^−1^ (R^2^ = 0.9955), respectively, in water (Fig 1 and Table 2). Correspondingly, their half-lives were 37.7 and 71.8 min, respectively, which was different from Sanchez-Prado’s report that the half-life time of BDE-47 undergoing aqueous photolysis was 4.16 min [28]. The *k* values for LBDEs increased with increasing bromine saturation in this study, which was in general agreement with that reported by Wei and coworkers [21]. Previous studies [11,17,29] have revealed that UVB and the shorter wavelength UVA radiation (ca. 310–320 nm) are responsible for the direct photochemical degradation of LBDEs in the environment. The difference of *k* values for LBDEs experiencing different radiation sources can be explained by greater overlap between the absorption spectra of the congeners and the mercury lamp radiation spectrum.

**Table 2:** Photolytic rate constants for PBDEs under 300 W mercury lamp irradiation (λ> 290 nm).

Reactions	*k* (×10^−3^)	
	BDE-28	BDE-47
Photolysis in ultrapure water	9.65	18.42
Photolysis in ultrapure water containing 100 mM IPA	9.01	17.52
·OH-induced photolysis in ultrapure water	0.64	0.90
Photolysis in ultrapure water containing 5 mM NaN_3_	7.34	14.05
^1^O_2_-induced photolysis in ultrapure water	1.67	3.47

The previous studies concluded that BDE-47 photolysis in organic solvents occurs through a reductive debromination mechanism and its rate is largely related to the capacity of the solvent to provide a hydrogen atom to the aryl radical formed by hemolytic cleavage of a C-Br bond after molecular excitation by radiation [4,11,17,27]. In general, a greater ability of the solvent to provide hydrogen promotes a higher rate for the photolytic process [11,30]. Eriksson and coworkers found that BDE-47 photolysis was slower in methanol/water (80:20) than in methanol [11], in agreement with the lower tendency of water to provide a hydrogen atom. The H-O bond dissociation energy in water is 119 kcal mol^-1^ while the C-H bond dissociation energy in water is 96 kcal mol^-1^ [31]. However, other reaction processes may occur in LBDEs photolysis. Xie et al. [30] observed a fast photodegradation of BDE-209 in CCl_4_ with a half-life only three times higher than that in THF, indicating that hydrogen-donating capacity of the solvent is not a major factor responsible for photolysis of BDE-209.

### Quantum yields of LBDEs in water

Quantum yield (Φ) is defined as the ratio between the amount of reactant consumed or product formed and the amount of photons absorbed. The Φ values for LBDEs photolysis was calculated using the following equation [32,33]:

$$\Phi_{substrate}=\;\frac{k_{substrate}\;\Sigma {\left(L_{\lambda }\varepsilon_{\lambda }\right)}_{actionmeter}}{k_{actionmeter}\;\Sigma {\left(L_{\lambda }\varepsilon_{\lambda }\right)}_{\;substrate}}\;\Phi_{actionmeter}$$ (3)

where Φ_actinometer_ is the known quantum yield of the *p*-nitroanisole/pyridine actinometer, *k*_substrate_ and *k*_actinometer_ are the rate constants for the disappearance of substrate and actinometer, L_λ_ values (lamp irradiance at a specific wavelength) were measured with a monochromator (Acton, SP-300) (S4 Fig), and *ε*_*λ*_ values are the molar absorptivities determined from UV/Vis spectra of the substrate (S5 Fig) or actinometer [33]. Because the solubility of LBDEs in water is very low, the determination of the molar absorptivity of LBDEs (10–20 μg L^-1^) in aqueous solution is very imprecise and presents a high associated error. To overcome this problem, solutions of LBDEs with different concentrations were dissolved in pure ethanol and in ethanol/water (80:20 and 20:80) and analyzed by UV-Vis spectrometry [24]. The molar absorbance of BDE-47 in different ratios of ethanol to water was similar to that reported by Eriksson et al. [11] in tetrahydrofuran (S5 Fig). There were no significant differences in molar absorptivities of BDE-28 between pure ethanol and the two ethanol/water ratios (80:20 and 20:80). The average molar absorptivity of BDE-47 and BDE-28 in the 290–340 nm wavelength range is shown in S6 Fig. According to Eq (3), the Φ values of BDE-47 and BDE-28 were 0.0479 and 0.0366, respectively. The calculated Φ values of BDE-47 in water was much lower than the values (0.22) determined by Eriksson et al. [11] in methanol/water; however, it was the same value (0.22) reported by Kuivikko et al. [17] in isooctane. Fang et al. [34] investigated the Φ values of 18 PBDE congeners substituted with 1–7 bromine atoms in hexane and ethanol under UV irradiation, and their values for BDE-47 and BDE-28 ranged from 0.12 to 0.19 in both hexane and ethanol. To the best of our knowledge, there is a paucity of data concerning quantum yield for PBDEs in water. According to Eriksson et al. [11] and Xie et al. [30], the capacity of the solvent to be a“hydrogen donor” substantially increases the photolytic rate and quantum yield of organic chemicals. The results of our study are consistent with these findings with our Φ values for BDE-47 and BDE-28 in water being much lower than those (0.12–0.19) in ethanol and hexane. In comparison, a highly polar solvent may quench the excitation state of organic chemicals, resulting in a low quantum yield [35]. As a result, the possible effect of the presence of acetonitrile in solution (0.1%) on LBDE photolysis should not be completely neglected. Acetonitrile present in solution can contribute to change the lifetime of the excited state or act as extra hydrogen source, changing the rate constant of LBDE photolysis. Thus, the calculated quantum yields of LBDEs in this work, although much more lower than the values in organic solvents [24], may still be overestimated.

### ROS-related indirect reaction mechanisms

#### ESR detection of ·OH and ^1^O_2_

ESR spectroscopy together with spin trapping techniques and the application of state-of-the-art loop gap resonators was used to provide a direct measure of spontaneous oxygen radical production by homogenates of freshly isolated and cultured rat pancreatic islets. The spin-trapping method with 5,5-dimethyl-1-pyrroline N-oxide (DMPO) and 2,2,6,6-tetramethyl-4-piperidone (TEMP) has been widely accepted to measure the ·OH and ^1^O_2_ scavenging activity of a compound in ESR spectroscopy, respectively. When the ESR signal of the DMPO-OH adducts decreases or disappears, it can be concluded that ·OH is scavenged by the additive, or that its formation is suppressed by the additive only by supporting observations [36]. Bektasoglu et al. [37] investigated the second-order reaction rate constant of ·OH and mannitol, an ·OH scavenger, was 0.6 × 10^9^ M^-1^ s^-1^. In the absence of TEMP, the ^1^O_2_ lifetime was 85 μs, while addition of TEMP shortened the ^1^O_2_ lifetime with *Kq* = 1.3 × 10^9^ M^-1^ s^-1^, suggesting TEMP could knock out the formation of ^1^O_2_ [38]. Oxidation of TEMP (2,2,6,6-tetramethylpiperidine) by ^1^O_2_ yields the TEMPO (2,2,6,6-tetramethyl-1-piperidinyloxyl) free radical easily detected by EPR (S7 Fig) [39].

**Fig 2:**
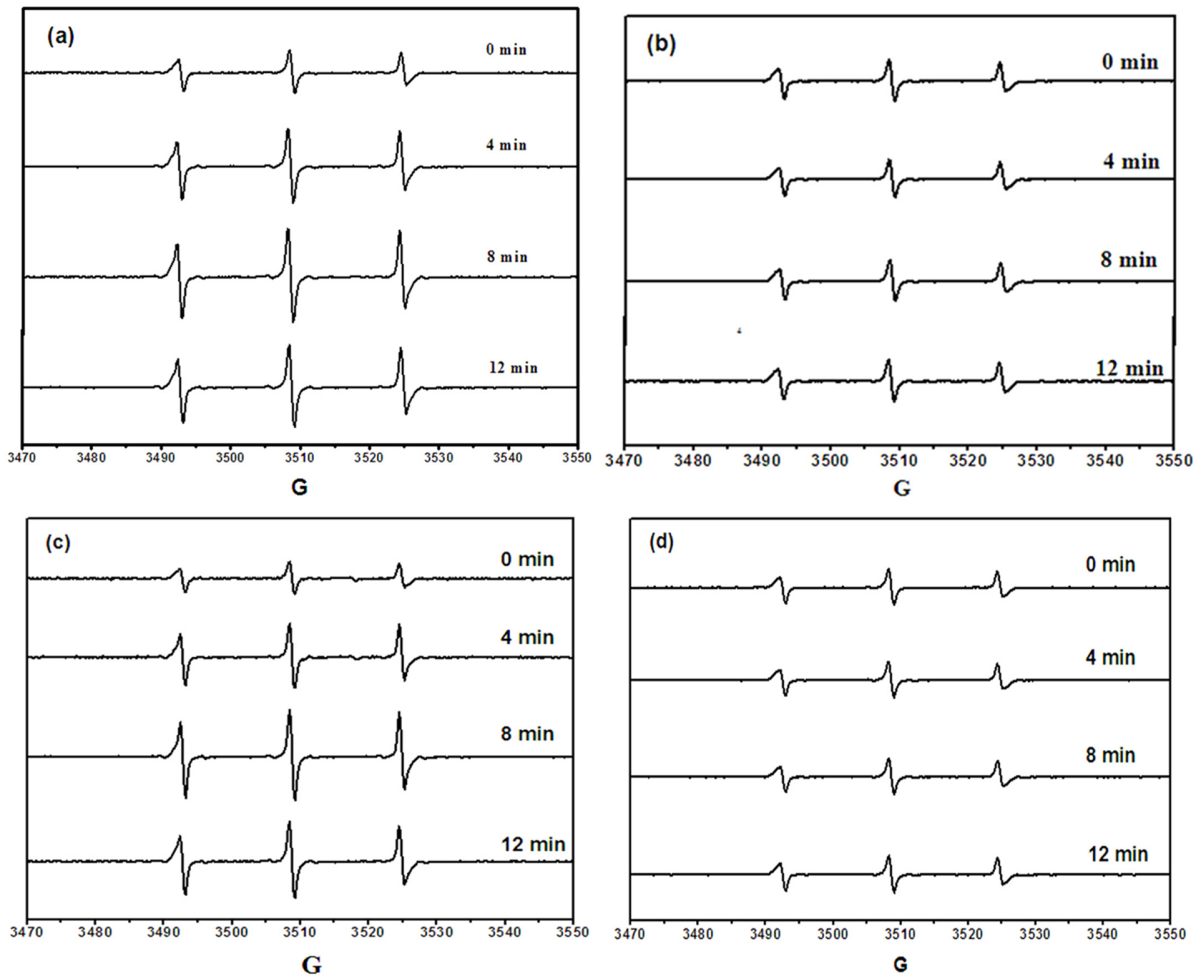
ESR spectra obtained at ambient temperature from the irradiation of LBDE solutions. Note: (1) The initial concentrations were 20 μg L^-1^ for LBDEs, 0.02 mol L^-1^ for TEMP, and 10 mM for sodium azide; (2) Irradiation time was 12 min; (3) Spectrum a and b for BDE-47; spectrum c and d for BDE-28.

ESR and spin-trap techniques were applied to probe singlet oxygen (^1^O_2_) and hydroxyl radicals (•OH) trapped by TEMP (0.02 M) and DMPO (0.05 M) [40–42]. ESR signals consisting of a 1:1:1 triplet were observed in the irradiation of LBDEs and 4-oxo-TMP solutions, which was attributed to the nitroxide radical adduct, 4-oxo-TEMP [40]. The intensity of the 4-oxo-TEMP signals rapidly increased with increasing irradiation time and reached a maximum after about 8 min of irradiation (Fig 2). The signal was then reduced from the maximum which was attributed to the disappearance of LBDEs when irradiated below 290 nm. To confirm that the formation of nitroxide was due to ^1^O_2_ reaction, NaN_3_ (sodium azide) was added to the LBDE and TEMP mixtures. The ESR signal of the nitroxide radical did not increase in the presence of NaN_3_ during the photolytic process (Fig 2b and 2d), which indicated that formation of the 4-oxo-TEMP spin adduct was greatly suppressed due to the quenching of ^1^O_2_ by NaN_3_.

**Fig 3:**
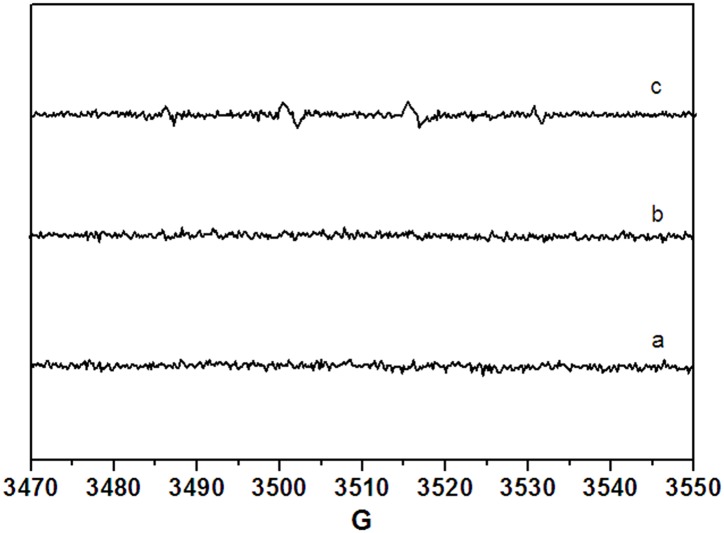
ESR spectra of ·OH spin-trapping with DMPO (0.05 mol L^-1^) from the irradiation of BDE-47 (20 μg L^-1^) solution in ultrapure water. Note: (a) in dark; (b) after 12 min of irradiation with addition of IPA (100 mM); (c) after 12 min of irradiation with DMPO.

ESR signals consisting of a 1:2:2:1 quartet pattern indicated that the DMPO-OH adduct was produced by irradiation of BDE-47 [43] (Fig 3c). However, only a weak DMPO-OH signal was observed after 12 min irradiation of BDE-47. When the BDE-47 solution was kept in the dark or irradiated with addition of isopropyl alcohol (IPA), no DMPO-OH signals were found (Fig 3b and 3c), which confirms that the ESR signal of DMPO-OH was formed via ·OH reaction. These results confirm that the photolysis of BDE-47 and BDE-28 involved both direct and indirect reaction mechanisms. Additionally, the indirect photolysis is dominantly attributable to ^1^O_2_ rather than ·OH [44]. In summary, we used ESR to demonstrate the formation of free radicals during the photodegradation of LBDEs in aqueous solutions.

#### Indirect photolytic contribution by ROS quencher

To further determine whether LBDEs underwent self-sensitized photo-oxidation via ROS, a series of quencher experiments were performed. IPA (100 mM) and NaN_3_ (10 mM), which were used to scavenge ·OH and ^1^O_2_, respectively, were added to the irradiated BDE-47 and BDE-28 solutions under 300 W mercury lamp irradiation. The addition of NaN_3_ in ultrapure water induced a pronounced inhibition of BDE-47 and BDE-28 photolytic rates under irradiation (λ>290 nm). In contrast, IPA had little effect on the photolytic rates of BDE-47 and BDE-28 (Fig 1). The contribution of indirect photolysis due to reaction with ·OH (*R*_•*OH*_) and ^1^O_2_ (RO12) during the LBDE photolysis process was calculated by Eqs (4) and (5):

$$R_{•OH}=\frac{k_{•OH\;(PW)}}{k_{PW}}=\frac{k_{PW}-k_{PW\;+\;IPA}}{k_{PW}}$$ (4)

RO12=    kO12 (PW)kPW=kPW + IPA−kPW + NaN3kPW (5)

As summarized in Table 2, *k*_*PW*_ *k*_PW+IPA_ and $k_{PW\;+\;NaN_{3}}$ describe the photolytic kinetics of LBDEs in pure water and with the addition of IPA and NaN_3_ in pure water, respectively. kO12 (PW) and *k*_•*OH*(*PW*)_ correspond to the photolytic kinetics for ^1^O_2_-induced and ·OH-induced LBDE photolysis, respectively [42,44]. Table 2 lists the reaction rate constants for BDE-47 and BDE-28 with different solution conditions. The indirect photolytic contribution rates in BDE-47 photo-oxidation process via ^1^O_2_ and ·OH were 18.8% and 4.9%, respectively. In comparison, those for BDE-28 were 17.3% and 6.6%, respectively. In the presence of IPA, the photolytic rate constants of BDE-47 and BDE-28 were 9.01 × 10^−3^ and 17.52 × 10^−3^ min^-1^, which were very close to those 9.65 × 10^−3^ and 18.42 × 10^−3^ min^-1^ in ultrapure water (without IPA). These findings further demonstrated that indirect photolysis of LBDEs was primarily attributable to ^1^O_2_ rather than ·OH.

#### Singlet oxygen reaction rate constants

To determine the rate constant for ^1^O_2_, the concentrations of LBDEs and FFA were measured during irradiation in the presence of RB as a photosensitizer for ^1^O_2_ production. FFA was recommended as a highly soluble, efficient trapping agent for singlet oxygen determinations in natural waters because its rose Bengal-sensitized photooxygenation was well documented [45,46]. The rate constant of the FFA reaction with singlet oxygen (k_FFA_) was determined to be 1.2 × 10^8^ M^-1^ s^-1^ [23,45]. The reaction produced 6-hydroxy(2H)pyran-3(6H)-one (pyranone) in an 85% yield [45] (S8 Fig). The loss of LBDEs by reaction with ^1^O_2_ was simultaneously monitored with that of the reference compound, FFA. The bimolecular reaction rate constant of LBDEs and ^1^O_2_ (k_rxn_) [47–49] was determined using the ratio of the slope obtained from a plot of substrate degradation *versus* that of FFA degradation as follows:

$$\text{ln}\frac{{\left[S\right]}_{t}}{{\left[S\right]}_{0}}=\frac{k_{rxn,s}}{k_{rxn,FFA}}\text{ln}\frac{{\left[FFA\right]}_{t}}{{\left[FFA\right]}_{0}}$$ (6)

**Fig 4:**
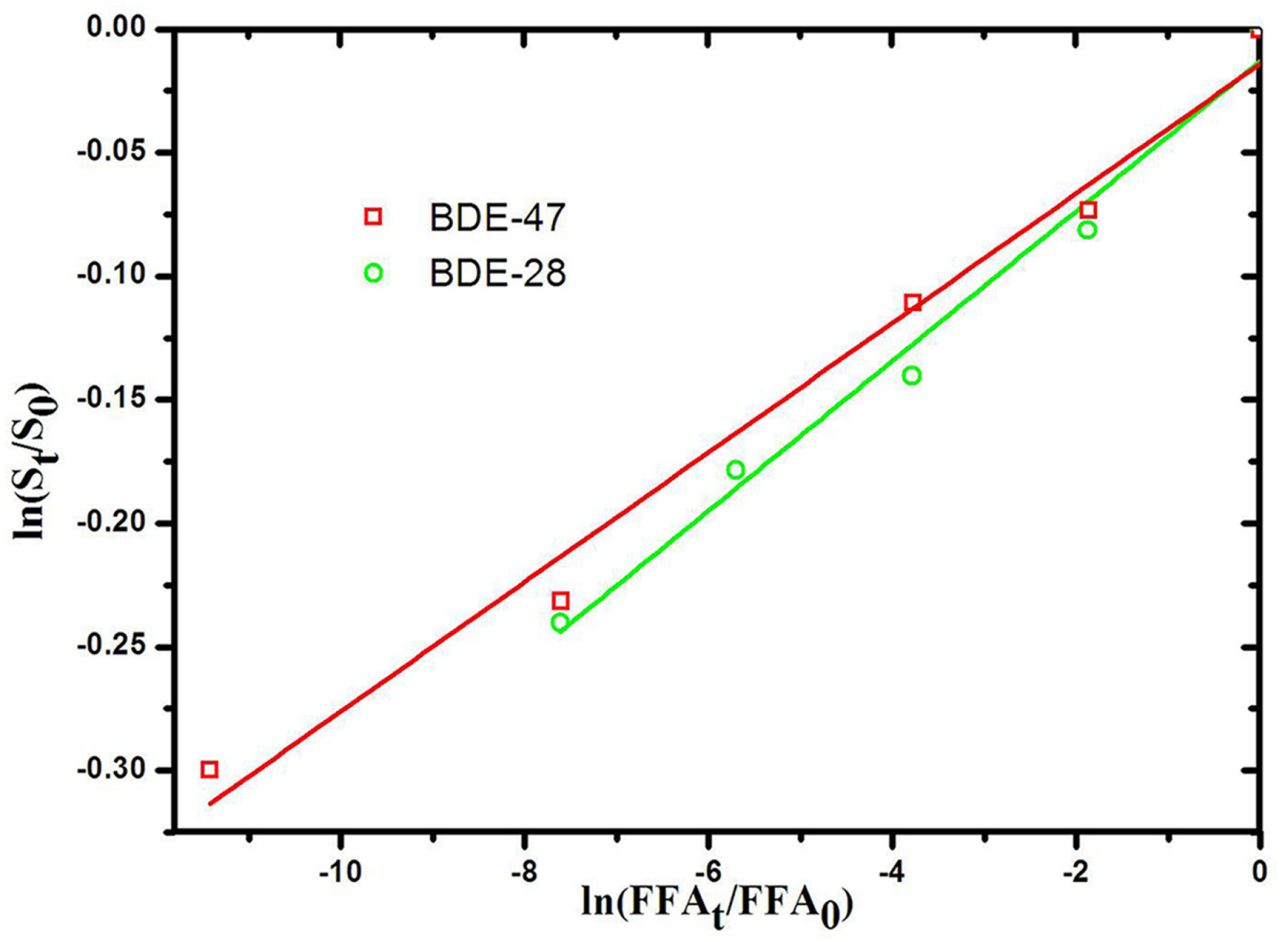
Loss of BDE-47 or BDE-28 *versus* loss of furfuryl alcohol in the presence of rose bengal in the solar simulator. Note: No direct photolysis in dark control.

The bimolecular reaction rate constants for ^1^O_2_ with BDE-47 and BDE-28 were 3.12±0.02 × 10^6^ M^-1^ s^-1^ and 3.64±0.04 × 10^6^ M^-1^ s^-1^, respectively (Fig 4). As reported in previous studies [49,50], the average ^1^O_2_ level in natural waters is ~10^−13^ M. Based on these data, the calculated *K* values for the reaction of ^1^O_2_ with BDE-47 and BDE-28 were 3.12 × 10^−7^ s^-1^ and 3.64 × 10^−7^ s^-1^, respectively. Tratnyek and coworkers found that an increase in chlorine substitution suppressed the singlet oxygen rate constants of chlorophenols [51]. Although the *k* value for the reaction of ^1^O_2_ with BDE-47 was a little lower than for BDE-28 in this investigation, further research is required to confirm if the *k* value is a function of the degree of bromine substitution with a corresponding suppression of the ^1^O_2_ rate constants for LBDEs.

#### Photolysis of BDE-47 in D2O aqueous solution

**Fig 5:**
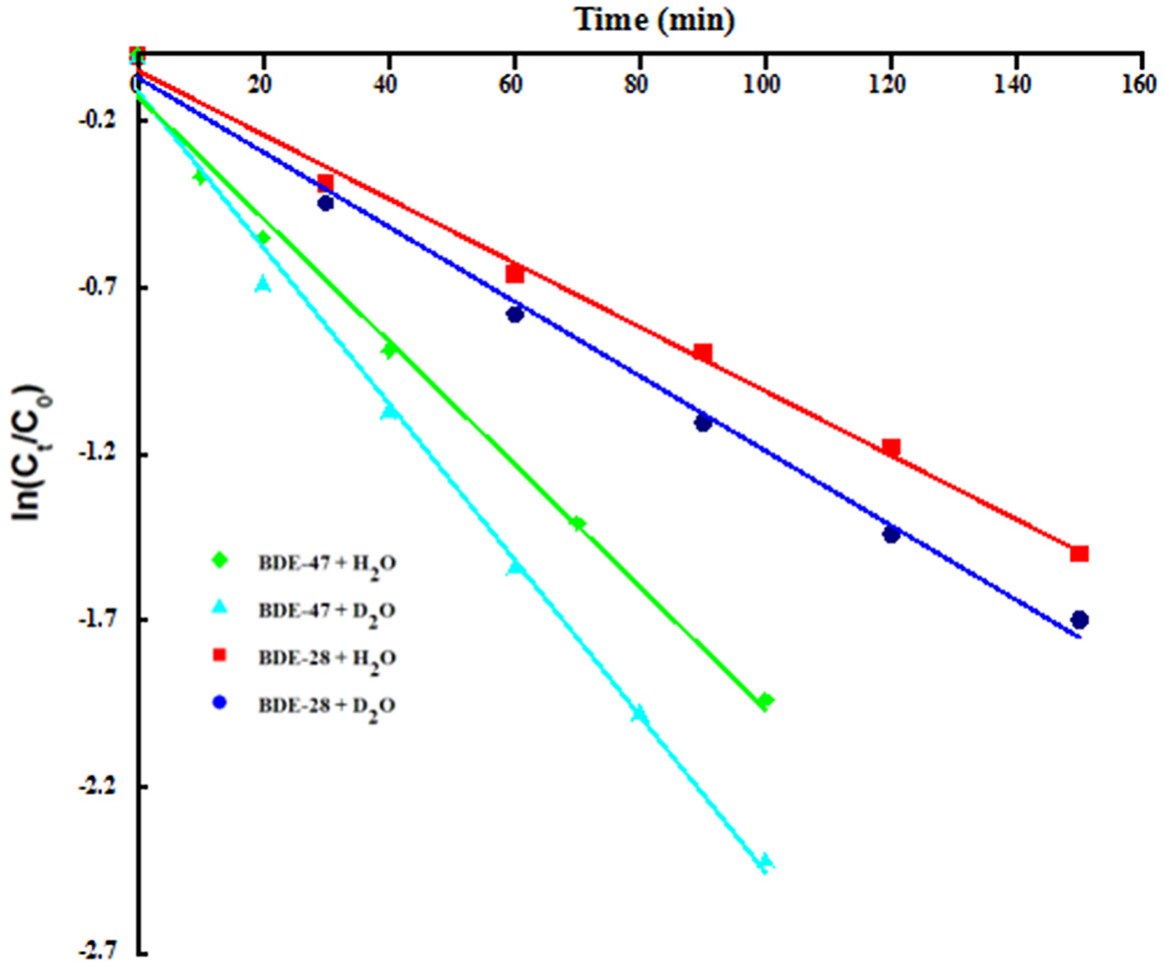
Effect of D_2_O on the photolytic rate of BDE-47 and BDE-28 in aqueous solutions under 300 W mercury lamp irradiation (λ> 290 nm).

Since ^1^O_2_ has a longer lifetime in deuterated solvents, reaction rates in 100% D_2_O will be faster than in 100% H_2_O if degradation is due entirely to ^1^O_2_ [52]. Therefore, if the reaction with ^1^O_2_ is the principal pathway for self-sensitized transformation of LBDEs, the rate constants should be significantly increased in D_2_O compared to H_2_O [53]. Under the above-mentioned experimental conditions, the degradation rate of BDE-47 in D_2_O showed enhancement by >20% when compared to that in H_2_O (Fig 5). The above results demonstrated that ^1^O_2_ is a major factor responsible for indirect BDE-47 photolysis, which was in agreement with the results obtained by NaN_3_ experiments.

## Supporting Information

S1 Fig Photochemical reactor system. The photodegradation experiments were performed in a quartz vessel with cover and magnetic stirring. A lamp (BiLon Corporation, Shanghai, China) equipped with cutoff filters was employed to provide irradiation. The 290 nm and 420 nm cutoff filters provided radiation in the range of 290 nm to 700 nm and 420 nm to 700 nm, respectively.
(TIF)

S2 Fig Dispersive liquid-liquid microextraction (DLLME) procedure. (TIF)

S3 Fig BDE-153 photoproducts at different irradiation times. (TIF)

S4 Fig Relative irradiance of the 300 W mercury lamp and the 300 W Xenon lamp and transmittance of the 290 nm cutoff filter. The light source irradiance spectra were measured with a monochromator (Acton, SP300).
(TIF)

S5 Fig Molar absorptivity coefficient of BDE-47 (a) and BDE-28 (b) as a function of wavelength in different proportions of ethanol: water (v/v). (TIF)

S6 Fig Molar absorptivity averages for BDE-47 and BDE-28. (TIF)

S7 Fig EPR signal of the TEMPO radical. (TIF)

S8 Fig Reaction of FFA and singlet oxygen. (TIF)

S1 Table The linearity and LOD for PBDEs assay. (DOC)
