# Supplementary figures and images for: Photolysis of Low-Brominated Diphenyl Ethers and Their Reactive Oxygen Species-Related Reaction Mechanisms in an Aqueous System
Source: PLoS One. 2015 Aug 14;10(8):e0135400. doi: 10.1371/journal.pone.0135400 (PMC4537200; doi:10.1371/journal.pone.0135400)

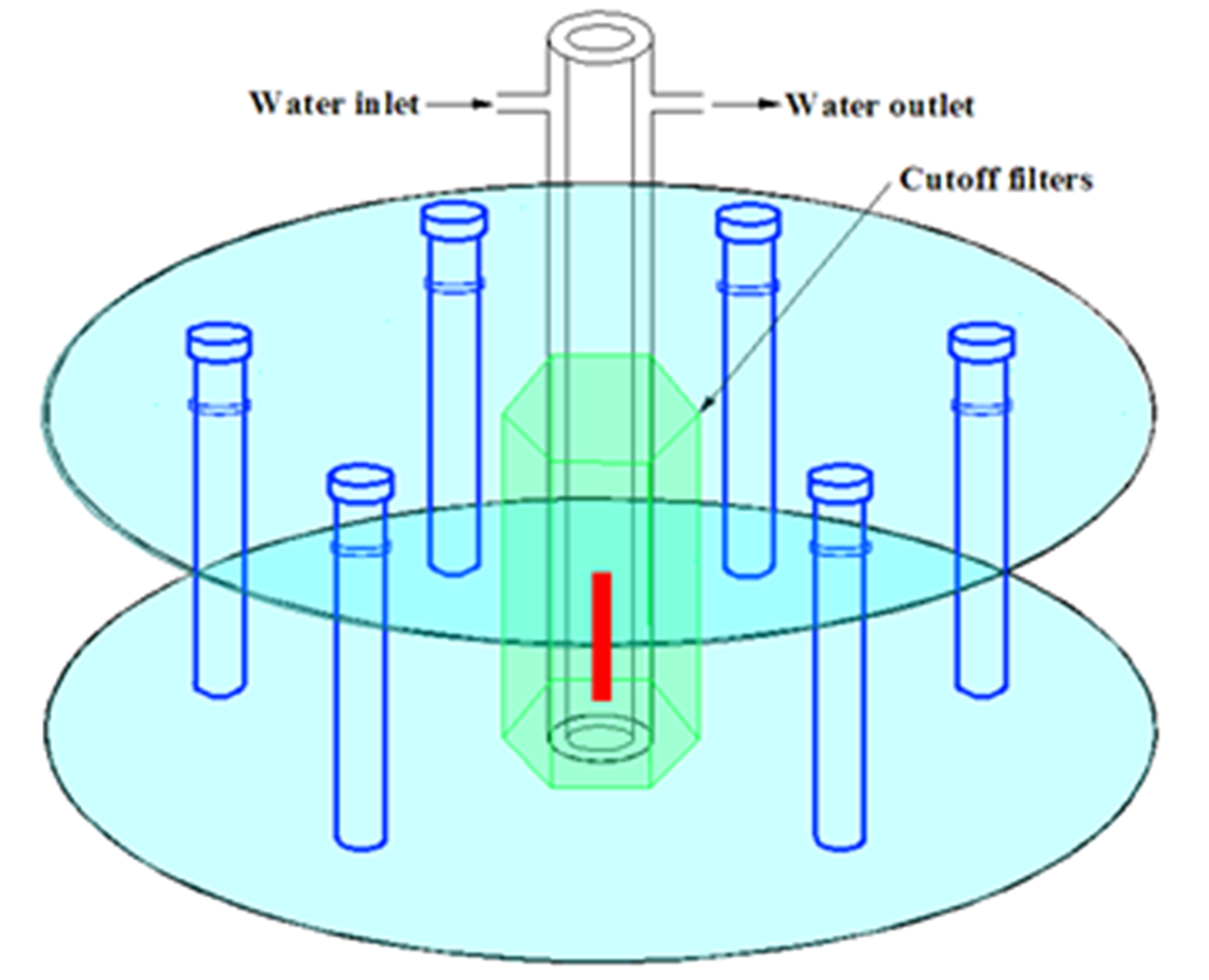

Supplement: S1 Fig — The photodegradation experiments were performed in a quartz vessel with cover and magnetic stirring. A lamp (BiLon Corporation, Shanghai, China) equipped with cutoff filters was employed to provide irradiation. The 290 nm and 420 nm cutoff filters provided radiation in the range of 290 nm to 700 nm and 420 nm to 700 nm, respectively. (TIF) [file pone.0135400.s001.tif]

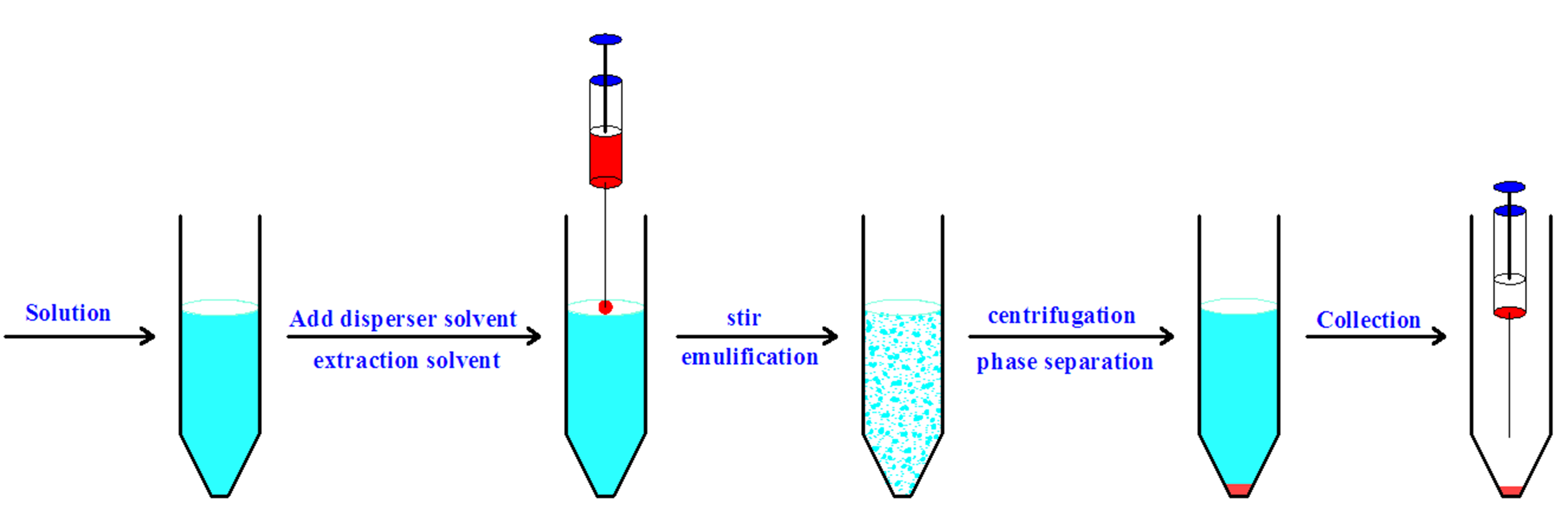

Supplement: S2 Fig — (TIF) [file pone.0135400.s002.tif]

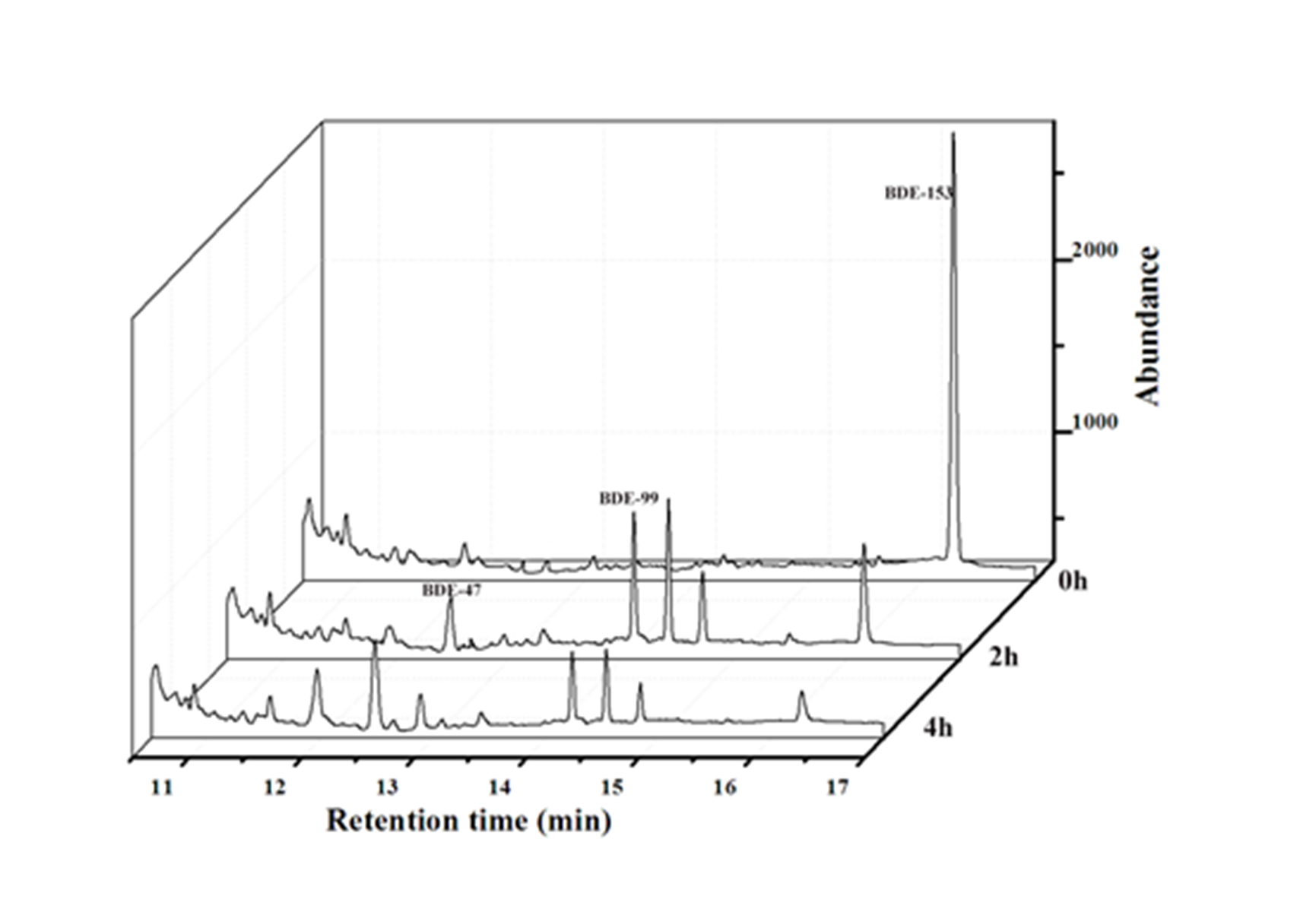

Supplement: S3 Fig — (TIF) [file pone.0135400.s003.tif]

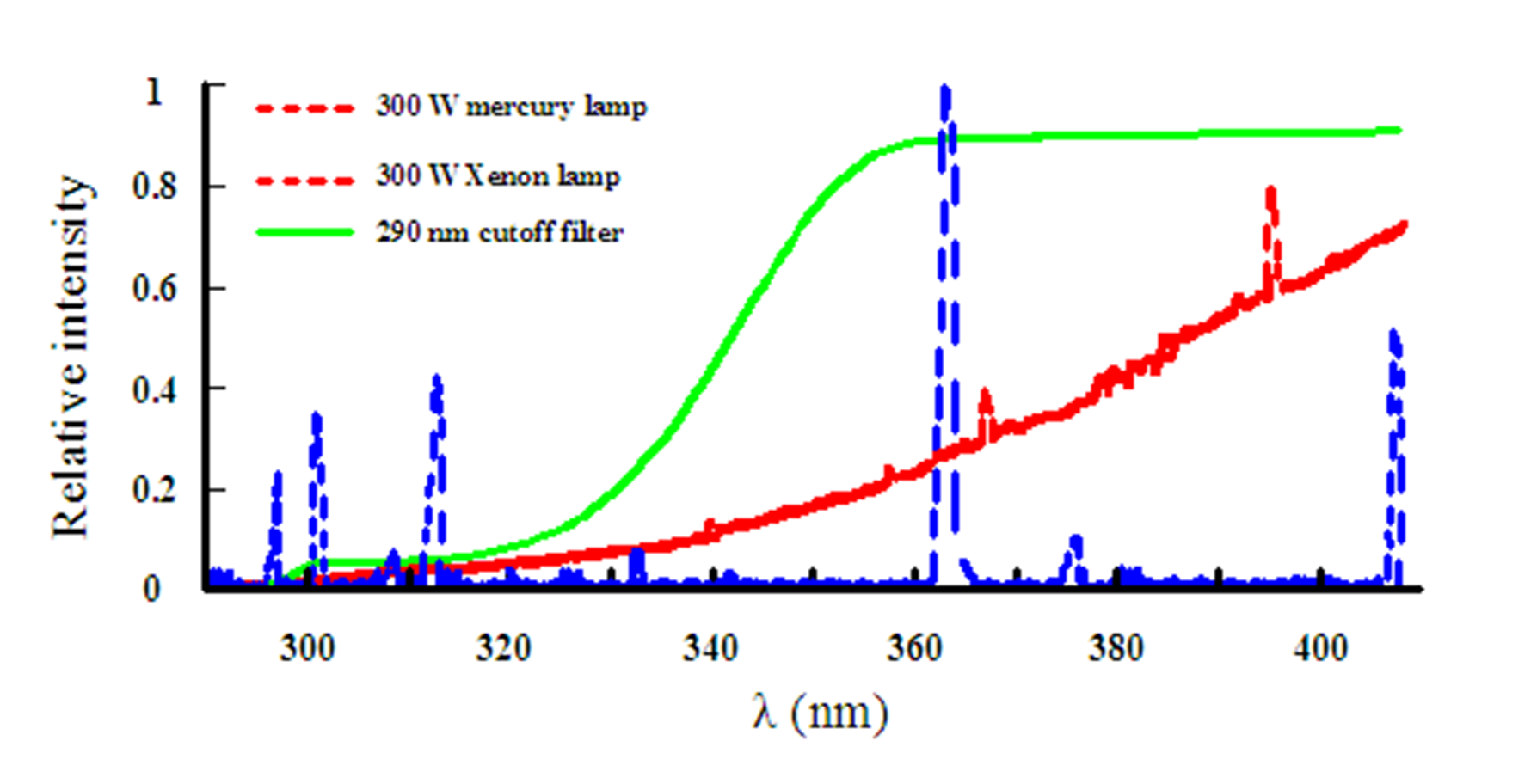

Supplement: S4 Fig — The light source irradiance spectra were measured with a monochromator (Acton, SP300). (TIF) [file pone.0135400.s004.tif]

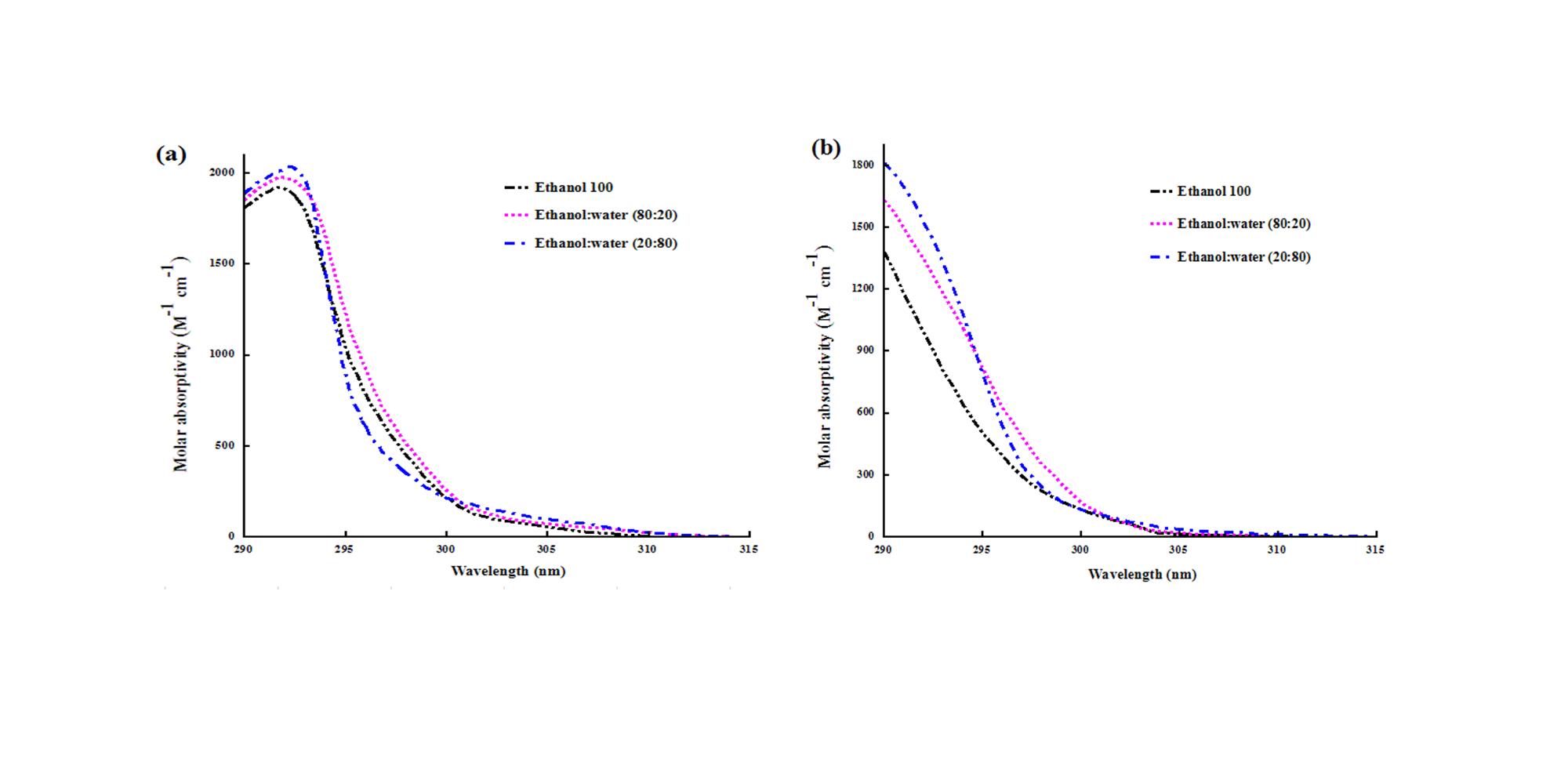

Supplement: S5 Fig — (TIF) [file pone.0135400.s005.tif]

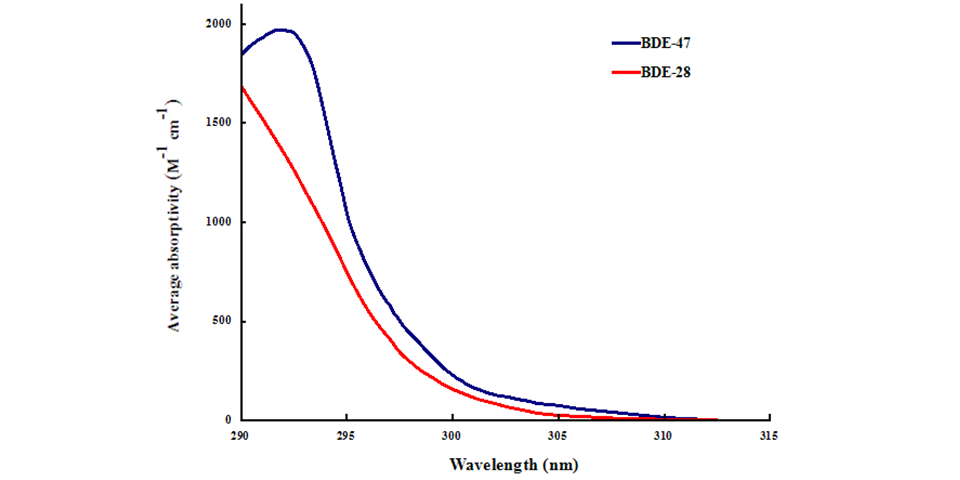

Supplement: S6 Fig — (TIF) [file pone.0135400.s006.tif]

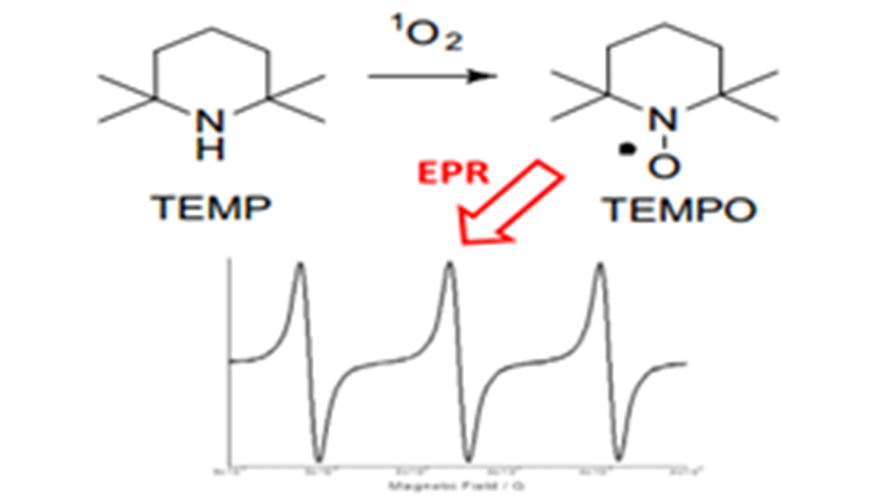

Supplement: S7 Fig — (TIF) [file pone.0135400.s007.tif]

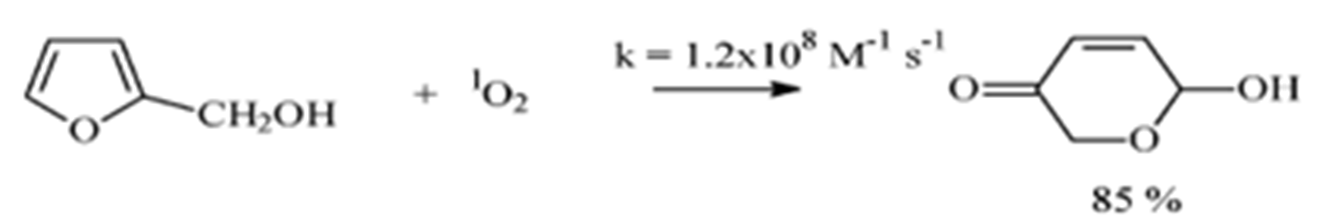

Supplement: S8 Fig — (TIF) [file pone.0135400.s008.tif]
