# Supplementary material for: Photolysis of Low-Brominated Diphenyl Ethers and Their Reactive Oxygen Species-Related Reaction Mechanisms in an Aqueous System
Source: PLoS One. 2015 Aug 14;10(8):e0135400. doi: 10.1371/journal.pone.0135400 (PMC4537200; doi:10.1371/journal.pone.0135400)
Supplement: S1 Table — (DOC) [file pone.0135400.s009.doc]

**S1 Table . The linearity and LOD for PBDEs assay.**

| Substance | Linear range (µg/L) | Linear correlation coefficients | LOD (ng/L) |
| --- | --- | --- | --- |
| BDE-28 | 0.1-50 | 0.993 | 8.1 |
| BDE-47 | 0.1-50 | 0.996 | 28.2 |
| BDE-99 | 0.1-50 | 0.992 | 22.5 |
| BDE-153 | 0.1-50 | 0.998 | 16.7 |
| BDE-154 | 0.1-50 | 0.993 | 13.4 |
| BDE-183 | 0.1-50 | 0.991 | 24.9 |
